# Serial interactome capture of the human cell nucleus

**DOI:** 10.1038/ncomms11212

**Published:** 2016-04-04

**Authors:** Thomas Conrad, Anne-Susann Albrecht, Veronica Rodrigues de Melo Costa, Sascha Sauer, David Meierhofer, Ulf Andersson Ørom

**Affiliations:** 1 Max Planck Institute for Molecular Genetics, Otto Warburg Laboratories, 14195 Berlin, Germany; 2 Department of Biochemistry, Free University of Berlin, 14195 Berlin, Germany; 3 Department of Informatics, Free University of Berlin, 14195 Berlin, Germany; 4 CU Systems Medicine, 97080 Würzburg 14195, Germany; 5 Max Planck Institute for Molecular Genetics, Mass Spectrometry Core Facility, 14195 Berlin, Germany

## Abstract

Novel RNA-guided cellular functions are paralleled by an increasing number of RNA-binding proteins (RBPs). Here we present ‘serial RNA interactome capture' (serIC), a multiple purification procedure of ultraviolet-crosslinked poly(A)–RNA–protein complexes that enables global RBP detection with high specificity. We apply serIC to the nuclei of proliferating K562 cells to obtain the first human nuclear RNA interactome. The domain composition of the 382 identified nuclear RBPs markedly differs from previous IC experiments, including few factors without known RNA-binding domains that are in good agreement with computationally predicted RNA binding. serIC extends the number of DNA–RNA-binding proteins (DRBPs), and reveals a network of RBPs involved in p53 signalling and double-strand break repair. serIC is an effective tool to couple global RBP capture with additional selection or labelling steps for specific detection of highly purified RBPs.

## Abstract (web summary)

RNA-binding proteins are involved in the posttranscriptional regulation of a large number of cellular processes and several recent studies have sought to describe the extent of the RNA-binding proteome. Here, Conrad *et al*. describe serIC, a stringent approach they apply towards defining the RNA-binding proteome of the mammalian nucleus.

## Graphical abstract

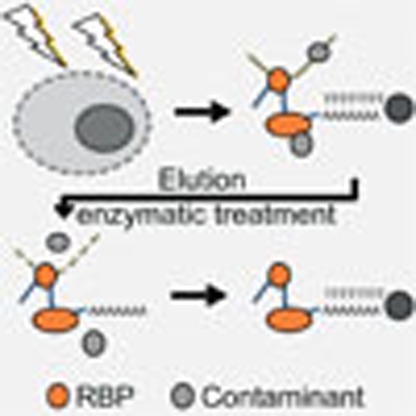

Advances in RNA research have revolutionized our understanding of RNA biology during the last decade^1^. It is now apparent that RNA molecules have regulatory and structural roles in virtually all cellular processes, functions that are executed via a largely unexplored dimension of RNA–protein interactions^2^,^3^. The vast interconnection between RNAs and protein factors is reflected in the coordinated cellular responses to external signals or insults. This includes the regulation of transcription, where the interplay of RNA and protein factors controls assembly of the transcriptional machinery at enhancers and promoters^4^,^5^. Accordingly, a growing number of dual specificity DNA–RNA-binding proteins (DRBPs) have been identified, although their exact number in the human genome is still unclear^6^. Further examples are the regulation of chromatin structure^7^, or the DNA damage response (DDR), where sensing of DNA damage and recruitment of DNA repair factors to lesions are paralleled by an extensive shift in RNA metabolism^8^,^9^. At the same time, several DNA repair proteins have the capacity to bind RNA, and an essential RNA component seems to be part of the DDR^10^,^11^,^12^,^13^.

The underlying protein–RNA interactions within such functional networks are still mostly deciphered on a gene-by-gene basis. Novel tools to accurately identify and characterize these interactions are therefore highly desired. Two landmark studies introduced the RNA interactome capture technique (IC), which for the first time enabled identification of *in vivo* protein–RNA interactions on a cellular scale^14^,^15^. This method uses irradiation of living cells with ultraviolet light to introduce covalent crosslinks between proteins and RNA in direct contact, while avoiding protein–protein crosslinks. After cell lysis, polyadenylated transcripts are captured by hybridization to oligo(dT)-beads, washed under denaturing conditions, and crosslinked proteins are identified by LC-MS/MS. The RNA interactomes presented by these global studies provided catalogues of RNA–protein networks in living cells. These networks were more extensive than previously assumed, comprising an unexpected number of proteins without classical RNA-binding domains (RBDs) or other features that allow the prediction of RNA-binding properties^14^,^15^. IC was subsequently applied to mouse embryonic stem cells and yeast, and consistently identified large numbers of proteins without assigned functions in RNA biology, collectively referred to as enigmRBPs^16^,^17^.

One limitation of previous global IC profiles is the lack of subcellular spatial resolution, which is especially relevant if the biological context of novel protein–RNA interactions is unknown. To catalogue the RNA–protein network involved in transcriptional regulation, nuclear RNA processing, chromatin structure and DNA repair, we thus set out to identify the nuclear RNA interactome. To enable comprehensive *in vivo* RBP detection with maximal specificity, we have developed serial IC (serIC), a method that includes repeated high-stringency RBP purifications. serIC can be easily coupled to intermittent enzymatic treatments or additional selection and modification steps to obtain highly selected RNA–protein complexes. When applied to isolated cell nuclei, serIC recovers substantially fewer proteins without predicted RNA-binding function than previous single-IC studies, and is thus in better agreement with computational RNA-binding predictions. With this nuclear RNA interactome we provide a high confidence list of dual specificity DNA–RNA binders, including transcription factors and chromatin components, with minimal background from DNA-binding proteins. Novel DRBPs extensively link transcription regulation with RNA processing. We also reveal a dense network of RNA-binding DNA repair factors involved in the p53 response and NHEJ, supporting the emerging concept of an RNA-guided DDR.

## Results

### serIC yields a highly purified nuclear RNA interactome

**Figure 1:**
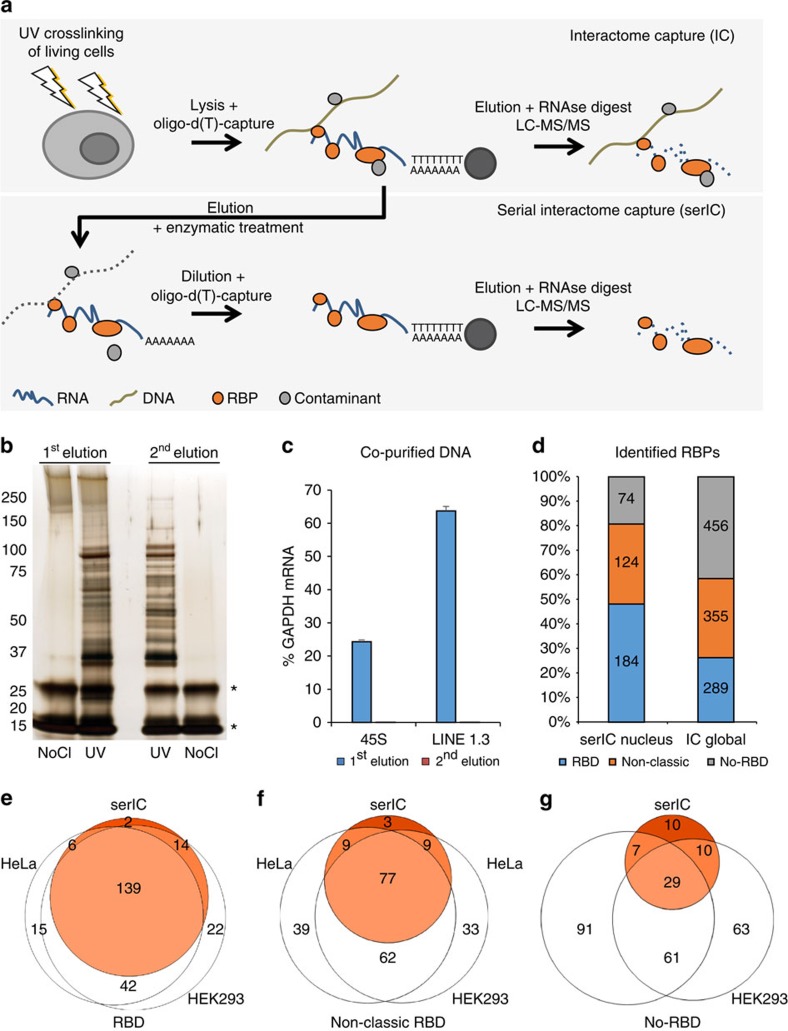
serIC recovers highly purified nuclear RBPs. (**a**) Comparison of the IC and serIC methods. Irradiation of living cells with ultraviolet (UV) light at 254 nm induces covalent crosslinks between proteins and nucleic acids. After cell lysis, RBP–poly(A)-RNA complexes are captured by hybridization to oligo(dT) magnetic beads and washed under denaturing conditions. In IC, purified material is eluted and proteins are identified by LC–MS/MS (top). In serIC, eluted material is enzymatically treated to remove contaminating DNA. Free RBP–poly(A)-RNA complexes are diluted in the presence of high salt and detergent to disrupt residual non-crosslinked interactions, recaptured by hybridization to oligo(dT) beads and analysed by LC–MS/MS. (**b**) SDS–PAGE of proteins crosslinked to poly(A)-RNA. Nuclei from irradiated K562 cells were isolated and subjected to the serIC procedure. Protein–RNA complexes obtained after the first or second round of purification (first elution and second elution) were digested with RNAseA/T1, separated on a polyacrylamide-gradient gel, and visualized by silver staining. No UV-crosslinking was performed in controls (No Cl). Asterisks indicate RNase A/T1. (**c**) Elimination of DNAse contamination. qPCR measurement of eluate from the first and second round of purification was performed without reverse transcription to detect residual DNA. Levels of 45S rDNA and LINE 1.3 retrotransposon DNA in the first and second eluate are shown relative to GAPDH mRNA in the second eluate. Error bars show s.d. from two independent experiments. (**d**) Domain composition of the nuclear RNA interactome. Proteins identified by LC–MS/MS in serIC preparations from K562 nuclei, and in previous IC from HeLa^15^ and HEK293 (ref. 14) were grouped by the presence or absence of classical or non-classical RBDs as indicated. (**e**–**g**) serIC recovery of proteins with the GO association ‘nucleus' that were previously identified by IC in HeLa^15^ or HEK293 (ref. 14). Proteins containing classical (**e**), non-classical (**f**), or no RBDs (**g**) were compared separately.

We initially set out to define a nuclear RNA interactome by applying IC to the nuclear and chromatin compartment of K562 myeloid leukaemia cells. We chose this cell line, since growth in suspension culture more easily allows scaling up starting material for IC by three- to fivefold compared with the two previous whole-cell studies, to compensate for the reduced material obtained from the nuclear compartment^14^,^15^. We irradiated ∼1 × 10^9^ K562 cells with ultraviolet light to induce protein–RNA crosslinks, and subsequently extracted whole nuclei or chromatin via biochemical fractionation^18^,^19^. Lysis, binding of poly(A)-RNA to oligo d(T) magnetic beads and subsequent washes were performed under denaturing conditions (see Methods; Fig. 1a)^14^,^15^. PAGE and silver staining reveal the specific recovery of crosslinked proteins (Fig. 1b, left panel). However, we detect a substantial amount of DNA in IC purifications, even after this stringent purification step (Fig. 1c). Residual DNA might be captured via RNA–DNA hybrids during the poly(A) pull-down, or via crosslinking of dual specificity DNA and RNA-binding proteins (RBPs). In either case, the presence of DNA in IC material is of concern, since this may lead to erroneous annotation of DNA-binding proteins as RNA binders. Elution of RBP–RNA complexes into low salt buffer enables subsequent enzymatic treatments of IC samples, which can be followed by a second round of oligo d(T)-capture and stringent washes prior to LC-MS/MS detection (Fig. 1a). We refer to this approach as serIC, and use it in conjunction with an intermittent turbo DNAse treatment to eliminate detectable DNA from purified poly(A)-RNA–protein complexes (Fig. 1c). At the same time, polyadenylated RNA is efficiently recovered during the serIC protocol (Supplementary Fig. 1a). Silver staining of isolated proteins reveals that a substantial amount of non-crosslinked background proteins is eliminated from non-crosslinked controls in serIC versus IC samples (Fig. 1b, right panel). Importantly, there are distinct changes in the recovered protein pattern in ultraviolet-crosslinked samples after serIC, indicating the removal of distinct contaminating proteins (Fig. 1b).

We finally subjected isolated proteins to LC-MS/MS. Proteins were identified using MaxQuant at an FDR of 0.01 (Supplementary Table 1)^20^. Gene ontology (GO) analysis of the identified proteins reveals a very high enrichment for the term ‘RNA binding' (corrected *P* value 9.5 × 10^−217^), confirming the specificity of the method (Supplementary Fig. 1b). Most identified proteins are associated to the GO term ‘nucleus', reflecting the nature of the starting material (Supplementary Fig. 1c). At the same time, the number of proteins associated to the term ‘cytoplasm' alone is strongly reduced compared with the previous IC studies from whole cells. As expected, proteins from chromatin purifications are largely recovered in serIC from whole nuclei (Supplementary Fig. 1d). In total, we identify 382 proteins by serIC in K562 nuclei and chromatin samples. This corresponds to about half the number identified in each of the global IC studies^14^,^15^,^16^,^17^, in line with the restriction to the nuclear compartment. For further analysis, we pooled the 382 RBPs and collectively refer to them as the nuclear RNA interactome.

### serIC recovers few unexpected RBPs

To further assess the structural features of the nuclear RNA interactome obtained by serIC, we grouped the identified proteins according to the occurrence of classical or non-classical RBDs as previously described^15^,^17^. We observe a pronounced shift in protein composition of the recovered RNA interactome (Fig. 1d). Forty-eight per cent of all proteins identified by serIC in K562 nuclei harbour a classical RBD, compared with only 26% in previous IC studies^14^,^15^. At the same time, the proportion of proteins without known RBD (no-RBD) is reduced by >50%. These differences remain if only annotated nuclear proteins are considered (Fig. 1e–h). Most nuclear proteins with classic RBDs that were previously identified in HEK293 and HeLa cells by IC are also detected by serIC (Fig. 1e)^14^,^15^. However, only about 42% and 18% of the previously identified candidates with non-classical or no RBDs, respectively, are detected after the serial purification (Fig. 1f,g).

**Figure 2:**
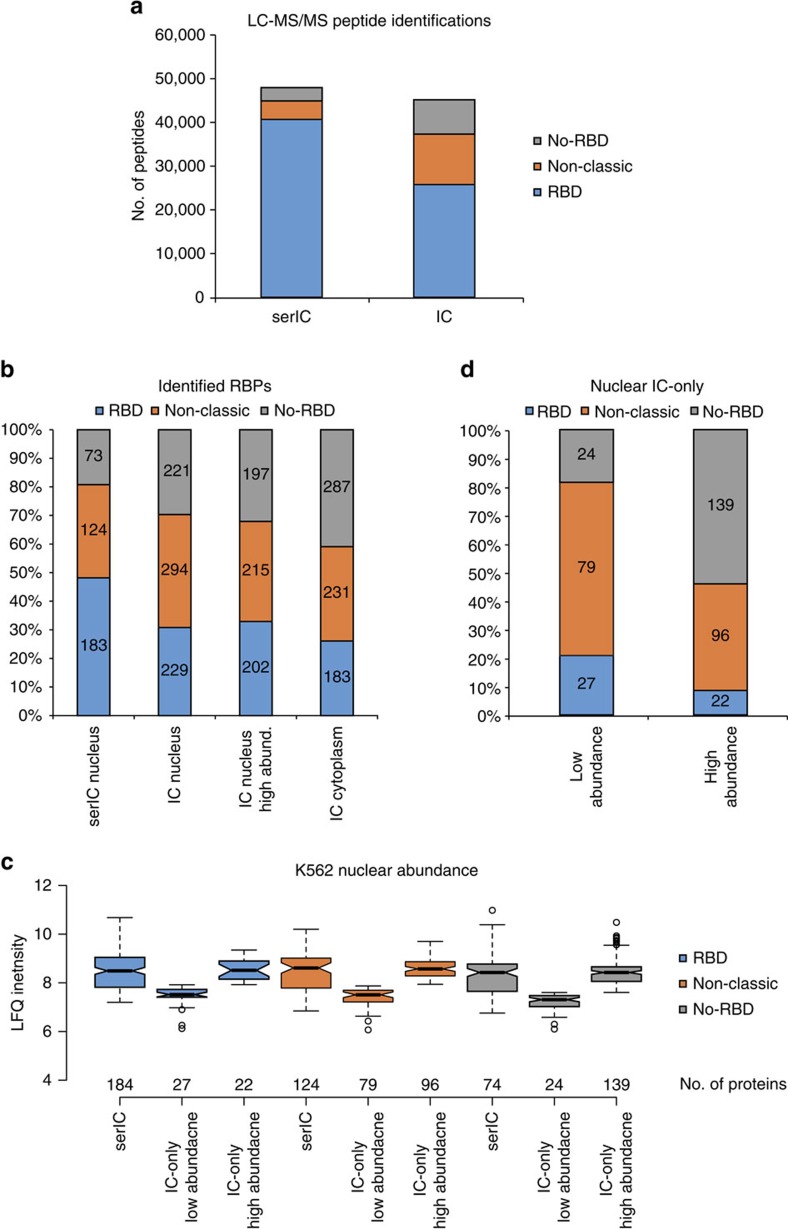
Altered domain composition of the serIC RNA interactome. (**a**) The combined number of individual LC–MS/MS peptide identifications in all serIC replicates and in all IC replicates from HeLa^15^. Indicated are peptides from proteins with classic, non-classic or no RBD. Only peptides from proteins that are enriched over the control are shown for each experiment. (**b**) Domain composition of the nuclear RNA interactome. Proteins identified by IC in HeLa^15^ and HEK293 (ref. 14) were filtered for expression in K562 nuclei or cytoplasm. All nuclear, only highly abundant nuclear, and cytoplasmic IC-derived candidates are included in the comparison, respectively. (**c**) K562 nuclear abundance of the RBPs detected by serIC or in IC only, as measured by LC–MS/MS^14^,^15^. IC-only candidates are subdivided into proteins with similar abundance as serIC-derived RBPs from the same class, and proteins with reduced abundance. (**d**) Distribution of lowly or highly abundant candidate RBPs. Highly abundant IC-only candidates mostly lack RNA-binding domains.

The disproportionate reduction of proteins lacking known RBDs in serIC experiments prompted us to compare the overall efficiency of the serIC purification strategy with previous IC data. Despite the constraint to the nuclear compartment, the total number of MS/MS peptide identifications is higher in serIC compared with the HeLa RNA interactome data^15^, demonstrating high purification efficiency and sensitivity of the experiment (Fig. 2a). Furthermore, fold enrichments of classic and non-classic RBDs are higher in serIC compared with the IC data from HEK293 cells, indicative of reduced non-specific background in serIC controls (Supplementary Fig. 2a)^14^.

We next asked whether the altered domain composition of the nuclear RNA interactome reflects differences in protein abundance between cell lines or between nuclear and cytoplasmic protein content. We therefore subjected K562 nuclear and cytoplasmic fractions to LC-MS/MS and identified 3,620 cytoplasmic and 2,225 nuclear proteins. For further comparisons, we restricted previous IC-derived proteins to members of this K562 proteome, and refer to them as candidate RBPs.

Comparison of the K562 nuclear versus cytoplasmic proteome reveals a similar number of RBD-containing proteins in both compartments, while the number of no-RBD proteins is higher in the cytoplasm (Supplementary Fig. 3). This difference is directly reflected in previous IC data, where more no-RBD proteins are found among cytoplasmic compared with nuclear candidate RBPs (Fig. 2b). The observed shift in domain composition between serIC and IC RNA interactomes persists if only nuclear candidate RBPs are included in the comparison (Fig. 2b). For each class of proteins, we further subdivided the IC-only nuclear candidates into a population with similar median abundance as the RBPs detected by serIC and a population with reduced abundance in K562 nuclei (Fig. 2c). The same shift in domain composition between serIC and IC-derived RNA interactomes is observed when only highly abundant nuclear candidate RBPs are considered (Fig. 2b).

The majority of nuclear IC-only proteins with classic RBDs is lowly abundant in K562 nuclei (Fig. 2d). Among the highly abundant IC-only proteins with non-classic RBDs are 42 ribosomal proteins, indicating reduced crosslinking of poly(A)-RNA to newly assembled ribosomal subunits in the nucleus. Most other non-classic IC-only candidates are also lowly abundant in K562 nuclei. Conversely, the vast majority of nuclear IC-only no-RBD candidates are found in the highly abundant group. At the same time, both IC-only and serIC no-RBD candidates are scattered across the full range of log2 fold enrichment values in the IC data (Supplementary Fig. 2b–g)^14^, suggesting that a more stringent fold-change cutoff does not improve the overlap between both methods.

Collectively, these data suggest that serIC efficiently detects RBPs on a global scale. The reduced number of identified no-RBD proteins does not appear to result from diminished purification efficiency, MS sensitivity or protein abundance, but is reminiscent of the observed reduction of unspecific interactions in serIC compared with single-IC purifications (compare Fig. 1b left and right panels).

### serIC recovers *bona fide* RBPs with high efficiency

**Figure 3:**
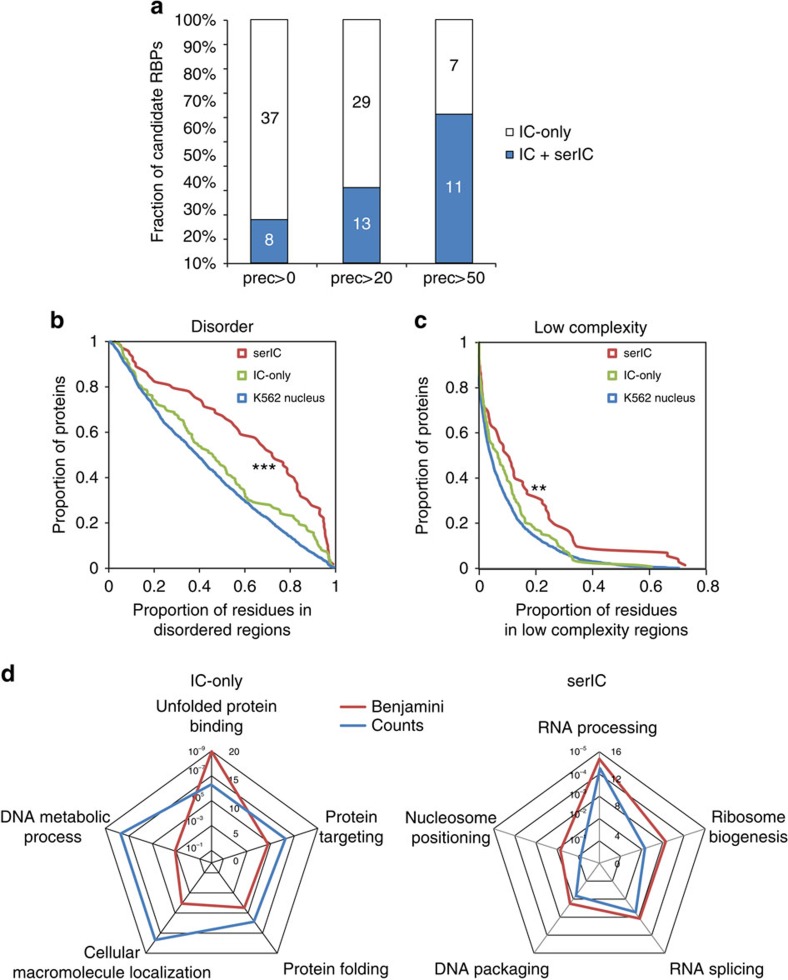
Good agreement between serIC and computational RBP prediction. (**a**) All nuclear no-RBD candidate RBPs detected by IC in HEK293, grouped by the precision of computational RNA-binding prediction^14^. Highlighted in blue are the proportion and number of proteins that are also identified as RBP by serIC. (**b**) Proportion of residues in disordered regions in all K562 nuclear proteins (blue), serIC-derived no-RBD proteins (red) and abundant IC-only nuclear no-RBD candidates (green). (**c**) Proportion of residues in low complexity regions with colour code as in **b**. Asterisks in **b** and **c** indicate *P* values <10^−3^ and<10^−2^, respectively. (**d**) GO-enrichment analysis of serIC-derived and IC-only nuclear no-RBD proteins. Shown are the most significantly enriched non-redundant ‘Biological Process' and ‘Molecular Function' GO terms compared with the human genome.

We next wanted to investigate how efficiently serIC can recover novel *bona fide* RNA binders. To test this, we used a computational RNA-binding prediction performed by Baltz *et al*.^14^ for the unannotated candidate no-RBD proteins identified by IC in HEK293. Here a multiple association network integration algorithm was applied to predict gene functions from Gene Ontology associations, domain composition, structural similarity, co-expression and protein–protein interaction data^14^,^21^. We grouped 105 highly abundant candidate no-RBD RBPs from Baltz *et al*.^14^ that are highly abundant in the K562 nuclear proteome according to the precision level at which RNA binding is predicted by the network integration algorithm. The precision of the RNA-binding prediction is defined as the proportion of genes that are correctly classified as having a given function, here RNA binding, estimated from applying the algorithm to a reference set of genes with known function^22^. Around 18% of the IC-derived nuclear candidate RBPs for which RNA binding could not be predicted with a precision above 0% are detected by serIC, confirming that the RNA interactome comprises some proteins that cannot be identified *in silicio* as RNA binders (Fig. 3a). Thirty-one and 61% of the nuclear no-RBD candidates with an RNA-binding prediction precision above 20% or 50%, respecitively, are recovered by serIC, highlighting the predictive power of the network integration algorithm used by Baltz *et al*.^14^, and confirming the sensitivity and accuracy of the serIC approach. Conversely, the fact that serIC is in better agreement with the computational prediction than single-IC highlights the improved specificity of RBP identification.

To further investigate whether no-RBD proteins identified by serIC are more likely to represent valid RBPs, and to gain further insight into the mechanisms of non-canonical protein–RNA interactions, we compared known structural features of RBPs between serIC and highly abundant IC-only no-RBD candidates. Unstructured and low complexity amino-acid sequences have been suggested to play an important role for mediating non-canonical protein–RNA interactions^15^,^23^. Accordingly, the proportion of amino acids within disordered regions is significantly higher in no-RBD proteins detected by serIC compared with IC-only candidates (*P*=1.4 × 10^−4^, Kolmogorov–Smirnov test), or to the K562 nuclear proteome (*P*≤10^−4^, Fig. 3b). Conversely, the distribution of disordered regions in IC-only proteins is statistically indistinguishable from the K562 nuclear proteome (*P*=0.26). Further corroborating this finding, no-RBD proteins detected by serIC display a significantly reduced amino-acid complexity compared with the K562 nuclear proteome (*P*=0.006), while complexity in IC-only candidates is indistinguishable from the background (*P*=0.1, Fig. 3c).

We furthermore assessed the functional properties of serIC versus IC-only no-RBD proteins. Figure 3d shows the most significant non-redundant GO terms for both groups. serIC-derived no-RBD proteins are highly associated with RNA-related biological processes. In contrast, IC-only candidates are enriched for RNA-unrelated processes like unfolded protein binding and DNA metabolism.

Several conserved protein domains show increased occurrences among serIC-derived no-RBD proteins, such as the Brix domain, which is found in proteins involved in ribosome biogenesis (Supplementary Fig. 4). Despite complete elimination of DNA contamination, the DNA-binding AKAP95 zinc finger and the conserved globular H15 domain from linker histones H1 are enriched among serIC no-RBD RBPs. At the same time, only the TCP-1/cpn60 chaperonin domain shows increased occurrence among abundant IC-only no-RBD candidates.. Collectively, the above analyses provide evidence for the capacity of the serIC approach to identify *bona fide* RBPs.

### serIC identifies dual specificity DNA–RNA binders

The group of dual specificity DRBPs is of particular interest, since rigorous elimination of DNA during the serIC procedure is expected to eliminate erroneous recovery of DNA-binding proteins. This is reflected by the elimination of 38 annotated DNA-binding proteins in serIC compared with the previous IC data. DRBPs are involved in various processes including the control of transcription, splicing and translation, DNA repair, cellular responses to stress and apoptosis. The extent of DRBPs in human is, however, still debated^6^.

**Figure 4:**
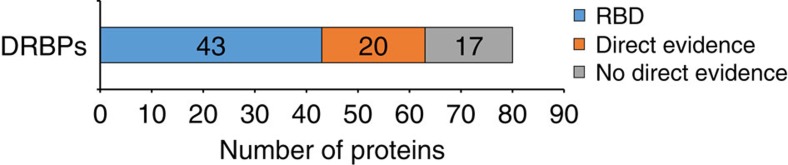
Novel DRBPs connect transcription regulation to the DDR. All annotated DNA-binding proteins identified in the nuclear RNA interactome. Proteins without classic RBDs were interrogated for previous direct evidence for RNA binding.

Overall, we identify 80 annotated DNA-binding proteins in the nuclear RNA interactome (Fig. 4). Forty-three of these contain classical RNA-binding domains. For another 20, direct RNA-binding has been demonstrated experimentally at the respective individual protein level. For 17 proteins, direct RNA binding has not previously been shown or has only been suggested by previous IC experiments where co-purification via DNA could not be excluded^14^,^15^. Ten of these novel DRBPs are transcription factors, seven of which have functions in RNA splicing or processing at the same time (Table 1). Three of these TF/processing factors are also involved in the DDR. We identify BCLAF and THRAP3, components of the SNARP complex, which binds to CyclinD1 messenger RNA and regulates its stability, but also couples the DDR to alternative splicing^24^. Two other SNARP components, PNN and SNW1, are also identified by serIC. The transcriptional repressor MYBBP1A is released from nucleoli to translocate to the cytoplasm and promote p53 acetylation when ribosomal RNA (rRNA) synthesis is inhibited. Due to the lack of an RBD it has been suggested that MYBBP1A is indirectly tethered to nucleolar rRNA^25^. Our data suggest that this regulation could be direct.

Several additional DRBPs couple transcription with RNA splicing. PHF5A binds to the CX43 promoter and stimulates its activation by oestrogen receptor alpha^26^. PHF5A is also part of the splicing factor 3b protein complex and has been suggested to bridge splicing factors and DNA helicases^27^. The AKAP95 domain containing ZNF326 can bind DNA to activate transcription, but is also part of the DBIRD complex that couples elongation by Pol II with alternative splicing^28^.

Two more DRBPs contain AKAP95 zinc fingers. AKAP8 binds the RII alpha subunit of PKA and regulates chromosome condensation^29^. An association of AKAP8 with the RNA helicase DDX5 has furthermore been observed at the nuclear matrix^30^. The paralog AKAP8L lacks the protein kinase A binding domain, but interacts with RNA helicase A^31^. RNA helicase interactions of both proteins may thus be related to their RNA-binding capacity.

We identify two kinases among the novel DRBPs. YLPM1 binds to the core promoter of TERT to control its downregulation in differentiating embryonic stem cells. YLPM1 has also been suggested to function together with PP1 as a modular nucleoside kinase, and to interact with the RBPs SAM68, NF110/45 and hnRNP-G in a nucleic acid-dependent manner, functions that are consistent with a direct RNA-binding capacity^32^. Another DNA–RNA binding kinase in the nuclear RNA interactome is PRKDC in the DNA-activated protein kinase (DNA-PK), the core regulator of DNA double-strand break repair by NHEJ^11^.

Finally, we find all four ubiquitous replication-dependent linker histones H1.2 to H1.5 but no core histones in the nuclear RNA interactome, suggesting an RNA-binding capacity of histone H1.

### serIC expands the network of RNA-binding DDR components

**Figure 5:**
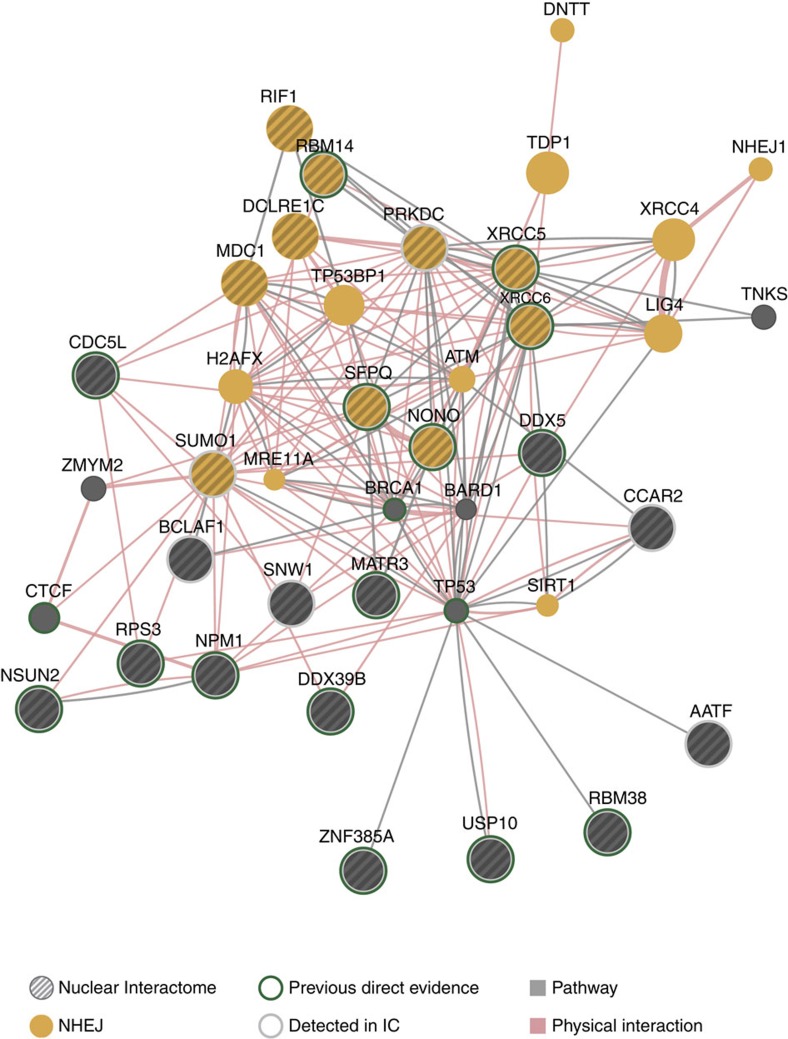
Network of DDR proteins in the nuclear RNA interactome. Nuclear RNA interactome proteins with GO annotations related to the DDR were used as query to construct a functional interaction network. First, neighbours were identified using the association data from protein and genetic interactions, pathways, co-expression, co-localization and protein domain similarity. Proteins were grouped based on physical and pathway interactions. Indicated are proteins involved in the NHEJ pathway and proteins with previous evidence for RNA binding.

The novel DRBPs identified by serIC support recent evidence for widespread crosstalk between RNA metabolism and the DDR^33^,^34^,^35^,^36^,^37^. In line with these findings, 22 genes with GO annotations related to the DDR are part of the nuclear RNA interactome, including several novel RBPs (Supplementary Table 1). To obtain an overview of the functional relationship between RNA-binding components of the DDR, we applied a multiple association network integration algorithm that uses information from protein–protein interactions; pathways; co-expression; co-localization; and shared protein domains to visualize interactions between the 22 query genes and their interacting proteins^21^. The resulting network is highly enriched for components of the DNA double-strand repair and the signalling cascades in response to DNA damage (Fig. 5). Two interacting factors, NSUN2 and MATR3, are also components of the nuclear RNA interactome. For 15 of the 24 identified RBPs in the network, previous evidence for RNA binding is available from direct assays on the individual protein level (green circles), while six additional factors have been suggested by IC (grey circles)^14^,^15^.

We identify 9 direct binding partners of p53 in the nuclear RNA interactome, including USP10, which deubiquitinates and activates p53 in response to DNA damage, and Sumo1, which can itself become covalently coupled to p53 to regulate its activity and subcellular localization (Supplementary Fig. 5)^38^,^39^,^40^. Several other members of the nuclear RNA interactome control p53 expression. CCAR2 is activated by ATM and ATR kinases to increase p53 transcription, and also regulates DNA repair at heterochromatic lesions^41^. Also AATF, which is stabilized upon phosphorylation by ATM, induces transcription of p53 and p53 target genes^42^. RBM38 in turn enhances translation of p53 and the cyclin-dependent kinase inhibitor p21 (refs 43, 44).

The most densely connected subnetwork of RNA interactome components is the NHEJ branch of the double-stranded DNA break repair pathway. Highlighted in Fig. 5 are factors that promote or are directly involved in NHEJ.

MDC1, a key component of the DDR, which we identify here for the first time as RBP, is recruited to phosphorylated yH2AX at DNA lesions, where it sustains and amplifies signalling by the ATM kinase together with TP53BP and provides a scaffold for the assembly of DNA damage *foci*^45^. MDC1 also directly interacts with the DNA-activated protein kinase (DNA-Pk), the core regulator of NHEJ and V(D)J recombination, and regulates its auto-phosphorylation in response to DNA damage^46^. It has been shown that recruitment of MDC1 to yH2AX *foci* is specifically disrupted on RNAse treatment, which is in agreement with an RNA-binding capacity^36^. We also identify RIF1, the main effector of TP53BP-mediated double-strand break repair pathway choice, as a novel RBP. RIF1 antagonizes BRCA1 and limits the recession of damaged DNA ends to prevent homology-directed repair (HDR) and directs the response towards NHEJ during G1 phase^47^,^48^,^49^,^50^. All components of DNA-PK, including the catalytic subunit PRKDC are also identified as part of the nuclear RNA interactome. Finally, Sumo1 functions in its conjugate form during classic NHEJ and as a free protein in Alt-NHEJ and HR^51^. We have validated the RNA-binding of four of these novel factors, THRAP3, BCLAF1, MDC1 and RIF1 (Supplementary Fig. 6). In addition to these novel factors, we recover known RBPs with functions in NHEJ. Taken together, serIC is effective in recovering a dense network of RNA-binding repair factors, suggesting tight cooperation of RNA and protein factors during the DDR.

## Discussion

Here we introduce serIC for global *in vivo* RBP identification with high specificity. We apply serIC to human cell nuclei to obtain global poly(A)-RNA–protein interactions with subcellular resolution. The resulting nuclear RNA interactome includes novel RBPs that link transcription, RNA processing, chromatin regulation and DNA repair, and will serve as a stepping-stone for further investigation of the underlying molecular mechanisms using gene-centered methods.

We observe a shift in domain composition of the nuclear RNA interactome compared with previous RBP catalogues derived by IC. The most substantial reduction is observed for previous candidate RBPs without known RBDs, despite high abundance of individual candidates in K562 nuclei, while observing no differences in purification efficiency or detection sensitivity of serIC compared with IC experiments^14^,^15^. Here one should consider that all purifications are performed under denaturing conditions so that detection of an RBP–poly(A)-RNA complex should solely depend on the *in vivo* crosslinking event, while the proportions of different RBP classes are not expected to change during more stringent washes. Conversely, more stringent purification procedures are expected to preferentially reduce the contribution from non-crosslinked non-specific protein interactions. In line with this, we observe non-crosslinked background proteins in non-irradiated controls after a single round of purification, which are eliminated after the second purification step of serIC. The enrichment of unfolded-protein binding chaperones in IC samples suggests that specific non-crosslinked interactions can be induced when captured RNA molecules are coupled to unfolded proteins in crosslinked samples, leading to false-positive RBP identification when comparing with non-crosslinked controls.

The distinct biological and structural features of the nuclear RNA interactome provide additional evidence for the high purity of RBP catalogues derived by serIC. No-RBD proteins detected by serIC are enriched for known features of classic RBPs compared with IC-only candidates, including a higher proportion of amino acids in disordered and low complexity regions^15^,^52^. At the same time, serIC-derived No-RBD proteins are enriched for RNA-related functions, while IC-only proteins are enriched for terms related to unfolded protein recognition, protein transport and DNA metabolism. Altogether, these results support the substantially increased specificity of serIC.

Coupling of serIC to intermittent enzymatic treatment is effective for the elimination of contaminating DNA, as evidenced by the removal of 38 DNA-binding proteins compared with IC. Elution of intact RBP–poly(A)-RNA complexes into low-salt conditions after each round of purification enables coupling of serIC to any additional isolation- or modification step, such as differential centrifugation, enzymatic treatment, labelling or affinity purifications. As long as these intermittent treatments leave RNA–polypeptide complexes intact, additional rounds of oligod(T)-capture can be performed.

Having ensured elimination of DNA from serIC samples, recovered DNA-binding proteins are likely to have dual DNA–RNA-binding specificities. Several modes of action have been proposed for DRBPs. DRBPs can bind DNA and RNA independently to execute multiple related or unrelated functions^6^. RNA and DNA interactions can also occur simultaneously, competitively or sequentially within the same biological process^6^. DRBPs detected by serIC extensively couple transcription factor activity with roles in RNA processing, which potentiates the capacity for gene expression regulation. This is exemplified by MYBBP1A, which interacts with the RNA Pol I complex to repress the transcription of rRNA genes, and at the same time interacts with the ribosome biogenesis machinery^53^. By ensuring efficient pre-rRNA processing MYBBP1 thus coordinates transcription and maturation of ribosomes. Our data suggest that MYBBP1 exerts this function via direct contact with its target gene product. Another interesting observation is the recovery of all replication dependent variants of Histone H1 by serIC. An RNA component has been shown to be involved in heterochromatin formation by HP1a^54^,^55^. At the same time, the chromodomain of HP1a cannot interact with native chromatin on its own, but is dependent on dynamic interactions with histone H1 to be functional^56^,^57^. It is tempting to speculate that the RNA-binding capacity of H1 takes part in this interplay.

A notable feature of the nuclear RNA interactome is the extended network of RNA binding DDR components. The identification of multiple regulators and effectors of p53 signalling and the recovery of the NHEJ module by serIC is in line with the emergent role of RNA biology in the DDR^33^,^34^,^35^,^36^,^37^. This interplay occurs at multiple levels: An extensive crosstalk has been observed between the DDR and posttranscriptional regulation of RNA splicing and stability, exemplified by the presence of BCLAF1 in the nuclear RNA interactome, which interacts with phosphorylated BRCA1 to connect the DDR with splicing regulation^58^. Recent evidence further suggests that ncRNAs are directly involved in the DNA repair process: The recruitment of MDC1 to DNA damage *foci* is RNAse sensitive and dependent on the local production of small RNAs that are generated in a Drosha and Dicer dependent manner^13^. The identification of MDC1 as RBP might provide a molecular basis for this observation. Since serIC recovers RBPs in complex with polyadenylated transcripts, it is tempting to speculate that components of the DDR can interact with such precursors. This would be in line with the observation that DDR proteins bind to Drosha and can control small RNA production^12^,^37^,^59^. Alternatively, long ncRNAs that are induced on DNA damage may provide a scaffold for the assembly of DDR proteins at DNA lesions, or act as decoys to sequester or dynamically regulate DDR factors during the multistep repair process^60^,^61^,^62^. The fact that we identify 10 RBPs that act sequentially and closely coordinated during NHEJ argues for such a scenario. Protein centered methods such as CLIP will help to identify interacting RNAs of individual DDR proteins and dissect the underlying molecular events.

Taken together, the first human nuclear RNA interactome presented here provides a resource for the future investigation of RNA-guided nuclear processes. In addition to the improved specificity provided by the serIC method, continuous refinement of experimental approaches and increasing MS sensitivity will further improve the ability to detect *bona fide* RBPs *in vivo*. At the same time, novel tools to globally recover non-polyadenylated RNA–protein complexes are needed to complete our picture of the RNA interactome. Accordingly, some aspects of the first RNA interactomes may have to be reassessed as we have certainly not yet unraveled the full extent of RBPs in the human genome.

## Methods

### Cell culture and nuclei isolation

K562 (ATTC) cells were grown in suspension culture at 75 r.p.m. in RPMI1640 medium supplemented with 10% fetal calf serum and antibiotics, and maintained at 37 °C and 5% CO_2_. For one replicate serIC experiment, 4 × 500 ml suspension culture were amplified to a density of 1 × 10^6^ cells per ml and collected by centrifugation at 200*g* and 4 °C. Cells were combined, washed with cold PBS, split in half and irradiated in a stratalinker with 0.4 J cm^−2^ UVC light (256 nm) or used as non-crosslinked control. Cells were recollected by centrifugation and resuspended in NP-40 lysis buffer (10 mM Tris pH 7.4; 150 mM NaCl; 0.15% NP-40; 1 mM DTT; complete protease inhibitors (Roche); 1/2,000 SUPERaseIn RNase Inhibitor (Thermo Fisher)). After 5-min incubation on ice, cells were gently layered over a cold sucrose cushion (10 mM Tris pH 7.4; 150 mM NaCl; 24% sucrose; 1 mM DTT) and centrifuged for 10 min at 1,000*g*. Nuclei were resuspended in IC lysis buffer (20 mM Tris pH 7.4; 500 ml LiCl; 1% LiDs; 5 mM EDTA; 5 mM DTT) and DNA was sheared by passing through a 20 1/2G needle.

For chromatin isolation, nuclei were instead resuspended in Glycerol buffer (20 mM Tris pH 7.4; 75 mM NaCl; 0.5 mM EDTA; 50% Glycerol; 1 mM DTT), mixed with Urea buffer (10 mM Tris pH 7.4; 7.5 mM MgCl2; 0.2 mM EDTA; 0.3 M NaCl; 1 M Urea; 1% NP-40; 1 mM DTT) and centrifuged for 3 min at 3,500*g*. The chromatin pellet was rinsed in cold PBS, snap frozen in liquid nitrogen, resuspended in chromatin lysis buffer A (20 mM Tris pH 7.4; 50 mM LiCl; 0.5% NP-40; 0.5% DOC; 0.1% SDS; 10 mM CaCl_2_; 10 mM MgCl_2_; 5 mM DTT; complete protease inhibitors (Roche)) and crushed by passage through a 10-ml syringe and 20 1/2G needle. After adding chromatin lysis buffer B (20 mM Tris pH 7.4; 600 ml LiCl; 1.2% LiDs; 6 mM EDTA; 5 mM DTT), the sample was filtered through a 100-μm cell strainer.

### Serial RNA interactome capture

*First purification*. The first part of the serIC purification was done largely as described by Castello *et al*.^15^. Eight ml oligo(dT) dynabeads (NEB) were equilibrated with IC lysis buffer (20 mM Tris pH 7.4; 500 ml LiCl; 1% LiDs; 5 mM EDTA; 5 mM DTT), and 2 ml were added to each tube with crosslinked or uncrosslinked lysate (∼100 ml crosslinked or non-crosslinked lysate from one biological replicate were each distributed into two 50-ml falcons). Samples rotated for 1 h at room temperature (RT) or overnight at 4 °C. Beads were collected on a magnet until the lysate was clear (30 min–1 h). We stored the supernatant at 4 °C for repeated purification, resuspended the beads in IC lysis buffer, rotated them for 5 min, and collected the beads with a magnet for 10 min. Beads were then washed twice in wash buffer 1 (20 mM pH 7.4 Tris HCl; 500 mM LiCl; 0.1% LiDS (wt/v); 0.1% NP-40; 1 mM EDTA; 5 mM DTT); twice in wash buffer 2 (20 mM pH 7.4 Tris HCl; 500 mM LiCl; 0.1% NP-40; 1 mM EDTA; 5 mM DTT); and twice in wash buffer 3 (20 mM pH 7.4 Tris HCl; 200 mM LiCl; 0.1% NP-40; 1 mM EDTA; 5 mM DTT). Finally, beads were collected in wash buffer 3 and transferred to a 1.5-ml eppendorf protein LoBind tube. RNA–protein complexes were incubated for 3 min at 55 °C in 1 ml elution buffer (20 mM pH 7.4 Tris HCl; 1 mM EDTA). Eluates were snap frozen and stored at −80 °C. This purification scheme was repeated twice with the lysate supernatant from above, yielding a total of 3 ml eluted material for UVC and No-Cl control. If possible, beads were reused each time after a brief wash in IC lysis buffer. We used fresh beads in case of extensive clogging after the elution step.

*Enzymatic treatment and second purification*. Triplicate eluates were pooled in 5-ml Eppendorf LoBind tubes. We added Turbo DNAse buffer and 40 μl Turbo DNAse; 40 μl superRNAsin; complete protease inhibitor; and incubated in 1.5-ml protein Lobind Eppendorf tubes at 37 °C for 20 min. Samples were transferred into falcon tubes and 3.5 ml fresh oligo (dT) dynabeads equilibrated in 12 ml IC lysis buffer were added. After 1 h rotation at RT, beads were collected on a magnet and the supernatant was kept on 4 °C for repeated purification. Beads were washed with IC lysis buffer for 5 min at RT, collected on a magnet and transferred to a 5-ml protein Lobind tube. Beads were washed twice with wash buffer 1; twice with wash buffer 2; and twice with wash buffer 3. After transfer to a 1.5-ml LoBind tube, RNA–protein complexes were incubated in elution buffer at 55 °C and 1,100 r.p.m. for 2 min. Beads were collected on a magnet, and the supernatant cleared on the magnet one more time. The cleared eluate was snap frozen and stored at −80 °C. Beads were resuspended in IC Lysis buffer and added back to the supernatant for two additional rounds of purification. All three eluates were pooled before downstream analysis.

### RNA and protein digestion

RNAse buffer (100 mM pH 7.4 Tris HCl; 1.5 M NaCl; 0.5% NP-40; 5 mM DTT) and RNAse A/T1 (200 U) were added to the remaining eluate and incubated for 1 h at 37 °C. One per cent of the material was used for SDS–PAGE and silver staining with the Pierce Silver Stain Kit (Thermo Fisher). The rest was used in LC-MS/MS.

### RNA and DNA analysis

To isolate RNA, 1% of the final pooled eluate was incubated with proteinase K buffer (50 mM pH 7.4 Tris HCl; 750 mM NaCl; 1% SDS; 50 mM EDTA; 2.5 mM DTT; 25 mM CaCl_2_) and 5 μg proteinase K for 1 h at 50 °C. RNA and DNA were isolated with Phenol chloroform. Reverse transcription was performed with and without RT-enzyme to monitor DNA contamination. RNA was reverse transcribed using the High-Capacity RNA-to-cDNA Kit (Invitrogen 4387406). cDNA was quantified on a 7900HT Fast Real-Time PCR system (Applied Biosystems) using the SYBR Green PCR Master Mix (Invitrogen 4364344).

Primer sequences:

45Sf: 5′- TCGCTGCGATCTATTGAAAG -3′

45S r: 5′- AGGAAGACGAACGGAAGGAC -3′

L1.3f: 5′- TGAAAACCGGCACAAGACAG -3′

L1.3 r: 5′- CTGGCCAGAACTTCCAACAC -3′

XIST f: 5′- CTCCACAATGCTTGCTCTGA -3′

XIST r: 5′- TAACCCCTGCCTGATAGGTG -3′

### LC–MS/MS

Samples for the serial RNA interactome capture were reduced in 50 mM DTT at 56 °C for 1 h and alkylated with a final concentration of 5.5 mM chloroacetamide for 30 min. Proteins were precipitated by four excess volumes of acetone at −20 C° ON. After two sequential acetone washes, lyophilized pellets were resuspended in 50 mM ammonium bicarbonate and 10% acetonitrile on a thermo rocker and proteins were digested by 0.75 μg trypsin at 37 °C ON. Peptides were desalted using C18 StageTips (Thermo Scientific, Waltham, MA) and lyophilized. 10% of each sample was used for direct LC–MS analysis, the rest was further separated using six pH fractions (pH levels 11, 8, 6, 5, 4 and 3) of strong anion-exchange chromatography (3 M Purification, Meriden, CT), according to ref. 63. Protein samples from the nuclear and cytosolic fractions were cleaned by centrifugal filtres (Amicon ultra-3K; Millipore, MA) and subsequently lysed and reduced in a 6 M guanidinium chloride buffer, digested by trypsin and fractionated by five strong anion-exchange chromatography fractions (SCX, 3 M Purification, Meriden, CT), according to ref. 64. Each strong anion-exchange chromatography or SCX fraction was dissolved in 5% acetonitrile and 2% formic acid and was injected for nanoflow reverse-phase liquid chromatography (Dionex Ultimate 3000, Thermo Scientific) coupled online to a Thermo Scientific Q-Exactive Plus Orbitrap mass spectrometer (nanoLC–MS/MS). Briefly, the LC separation was performed using a PicoFrit analytical column (75 μm ID × 25 cm long, 15 μm Tip ID (New Objectives, Woburn, MA) packed in-house with 3 μm C18 resin (Reprosil-AQ Pur, Dr Maisch, Ammerbuch-Entringen, Germany) under 50 °C controlled temperature. Peptides were eluted using a nonlinear gradient from 3.8 to 40% solvent B in solvent A over 180 min at 266 nl min^−1^ flow rate. Solvent A was 0.1% formic acid and solvent B was 79.9% acetonitrile, 20% H2O, 0.1% formic acid). Nanoelectrospray was generated by applying 3 kV. A cycle of one full Fourier transformation scan mass spectrum (300−1,700 *m/z*, resolution of 35,000 at *m/z* 200) was followed by 12 data-dependent MS/MS scans with a normalised collision energy of 25 eV. A dynamic exclusion window of 30 s was used to avoid repeated sequencing of the same peptides. MS data were analysed by MaxQuant (v1.5.0.0)^20^, and searched against the ENSEMBL release GRCh37.70 human proteome database, or UniProtKB with 69,714 entries released in 06/2015, using an FDR of 0.01 for proteins and peptides with a minimum length of seven amino acids. A maximum of two missed cleavages in the tryptic digest was allowed. Cysteine carbamidomethylation was set as a fixed modification, while N-terminal acetylation and methionine oxidation were set as variable modifications. Ion counts from two biological replicates were pooled and a threefold ratio of ultraviolet versus non-crosslinked control was used as cutoff after replacing zero counts in one condition with a pseudocount of one. LFQ, a generic method for label-free quantification^65^ within MaxQuant, was used for allocating proteins from nuclear and cytosolic SCX fractions.

### GO and domain analysis

GO annotations, Pfam and Interpro Domain annotations for the identified proteins were retrieved from the geneontology website and using ENSEMBL Biomart and the Ensembl Human release 81 (GRCh38.p3). Proteins were grouped for the presence of classic or non-classic RNA binding domains as in ref. 15. In brief, proteins were first scored for the presence of at least one classic RBD, the remainder was scored for the presence of at least one non-classic domain, and the rest were regarded no-RBD proteins. Enrichments of domains and GO terms were calculated using the DAVID tool. Corrected *P* values were obtained by the method of Benjamini–Hochberg.

### Disorder and low complexity

For each amino-acid position in a protein a score for intrinsic disorder was derived using IUPred^66^. Positions wit a score <0.4 are regarded disordered, and the proportion of these residues is calculated for each protein. Complexity was calculated as the Shannon entropy in a window of ±10 amino acids around each amino-acid position in a protein, and the proportion of positions with an entropy below 3 bit was derived for each protein. Differences in the distributions of disorder and low complexity regions were tested for significance using the Kolmogorov–Smirnow test.

### Interaction networks

PPI interaction networks were calculated using the GeneMANIA algorithm. We used the full information provided by the GeneMANIA database, including 287 co-expression data sets, 3 co-localization data sets, 7 genetic interaction data sets, 7 pathway collections, 190 data sets with physical interactions and 42 interaction prediction data sets were used to identify the 20 next neighbouring proteins of the 22 DDR-related query genes. Only physical and pathway interactions were used to visualize the network map.

## Additional information

**Accession codes:** The mass spectrometry proteomics data have been deposited to the ProteomeXchange with the dataset identifier PXD003664.

**How to cite this article:** Conrad, T. *et al*. Serial interactome capture of the human cell nucleus. *Nat. Commun.* 7:11212 doi: 10.1038/ncomms11212 (2016).

## Supplementary Material

Supplementary Information Supplementary Figures 1-6 and Supplementary Methods

Supplementary Table 1 Overview of identified proteins in noCl and UV treated samples

## Figures and Tables

**Table 1:** Functional classification of novel DRBPs identified in serIC.

**Protein**	**TF**	**RNA processing**	**Splicing**	**DDR**	**Enzyme**
THRAP3	×	×	×	×	
BCLAF1	×	×	×	×	
ZNF326	×	×	×		
PHF5A	×	×	×		
MYBBP1A	×	×		×	
BDP1	×	×			
AATF	×			×	
YLPM1	×				×
CEBPZ	×				
KDM5A	×				×
PRKDC				×	×
AKAP8					
AKAP8L			Putative		
HISTH1B					
HISTH1C					
HISTH1D					
HISTH1E					

DDR, DNA damage response; DRBP, DNA–RNA-binding protein; serIC, serial RNA interactome capture; TF, transcription factor.
