# Supplementary material for: Serial interactome capture of the human cell nucleus
Source: Nat Commun. 2016 Apr 4;7:11212. doi: 10.1038/ncomms11212 (PMC4822031; doi:10.1038/ncomms11212)
Supplement: Supplementary Information — Supplementary Figures 1-6 and Supplementary Methods [file ncomms11212-s1.pdf]

Supplementary Figure 1

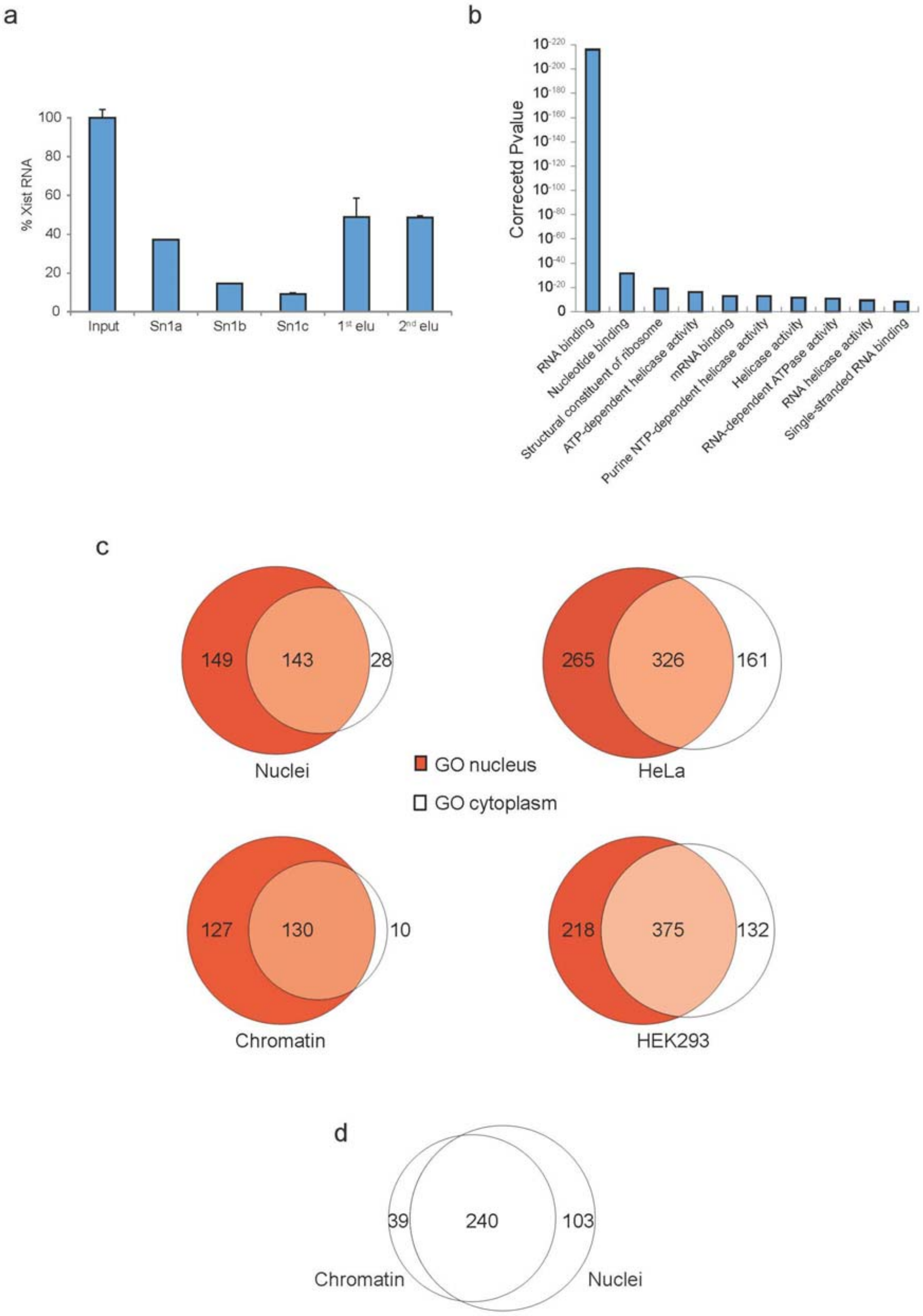

Supplementary Figure 1: GO analysis for proteins identified in serIC.

(**a**) pPCR analysis of Xist RNA in K562 nuclear extract (Input), supernatants (Sn1a to Sn1c) and pooled eluate from the first round of purification (1st elu); and the pooled eluate from the second round of purification (2nd elu). Levels are normalized to Xist RNA in the Input. Error bars show s.d. (**b**) Shown are the 10 most highly enriched “Molecular Function” GO terms for all enriched proteins compared to the human genome. (**c**) Number of proteins with GO annotation “nucleus” or “cytoplasm” in serIC experiments from chromatin or nuclei; and from HeLa and HEK293 IC experiments [18,19]. (**d**) Overlap between serIC protein identifications from chromatin and nuclear samples.

Supplementary Figure 2

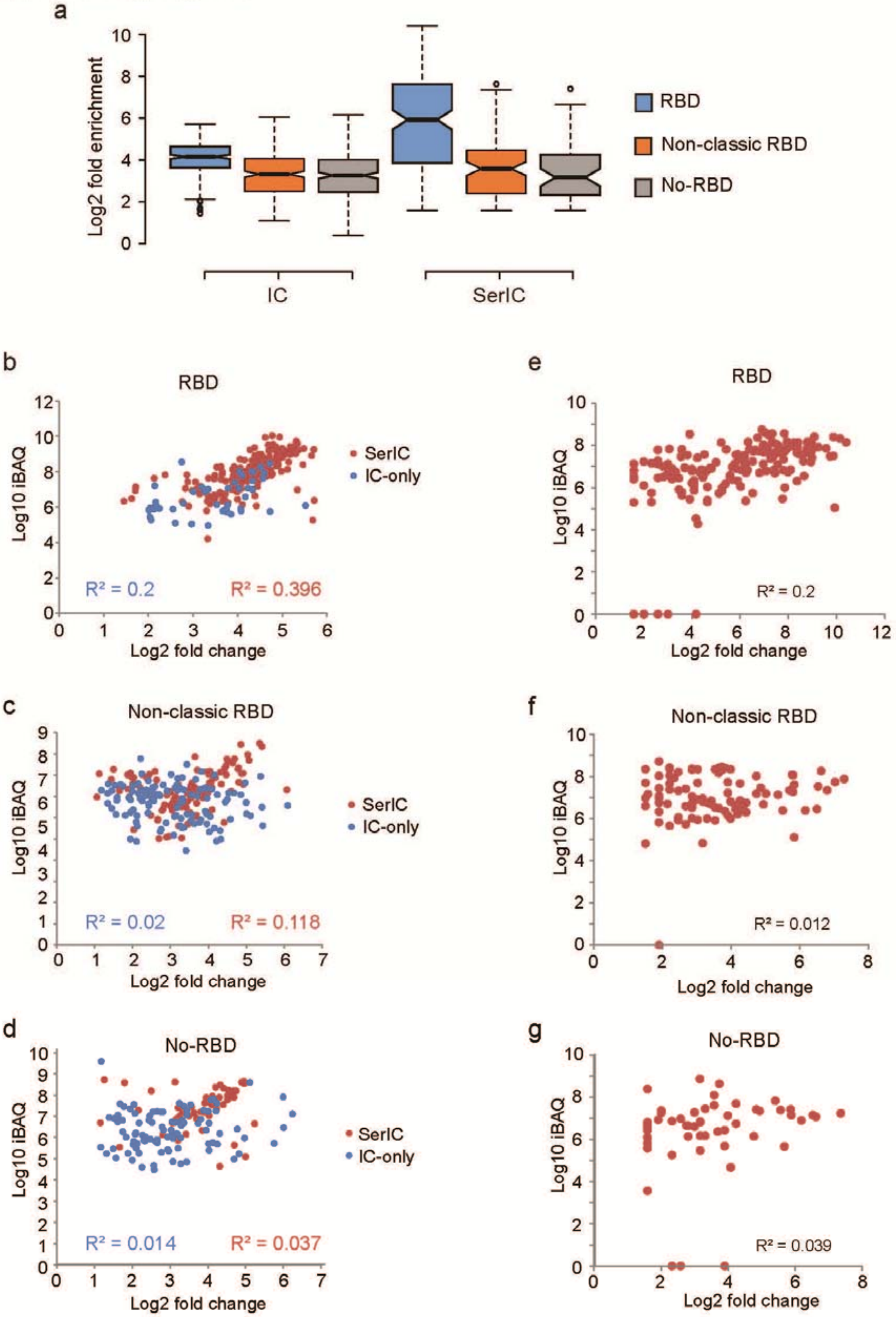

Supplementary Figure 2: Comparison of serIC and IC fold enrichments.

(**a**) Fold enrichment values from HEK293 IC data [18] and from serIC were grouped by the presence or absence of classical or non-classical RBDs as indicated. (**b-d**) Shown are protein abundances and fold recoveries of RBPs detected by IC in HEK293 [18] for classic (**b**), non-classic (**c**) and no-RBD candidates (**d**) that are present in K562 nuclei and recovered (red) or not recovered (blue) by serIC. (**e-g**) Shown are protein abundances and fold recoveries of RBPs detected by serIC in K562 nuclei for classic (**e**), non-classic (**f**) and no-RBD proteins (**g**).

Supplementary Figure 3

a

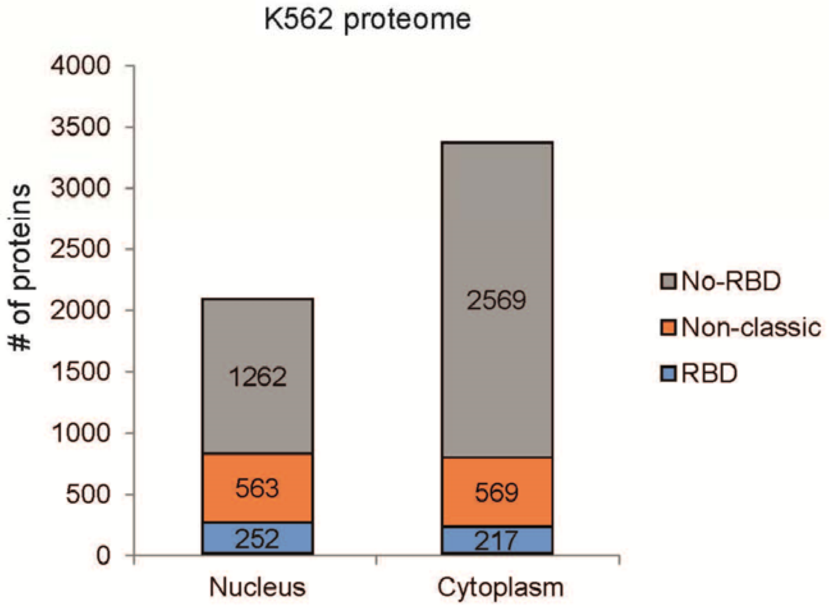

Supplementary Figure 3: Occurrence of RBDs in the cytoplasm and nucleus of K562 cells.

(**a**) Proteins identified in the cytoplasm or nucleus of K562 cells by LC-MS/MS were grouped by the occurrence of classic or non-classic RBDs as indicated.

Supplementary Figure 4

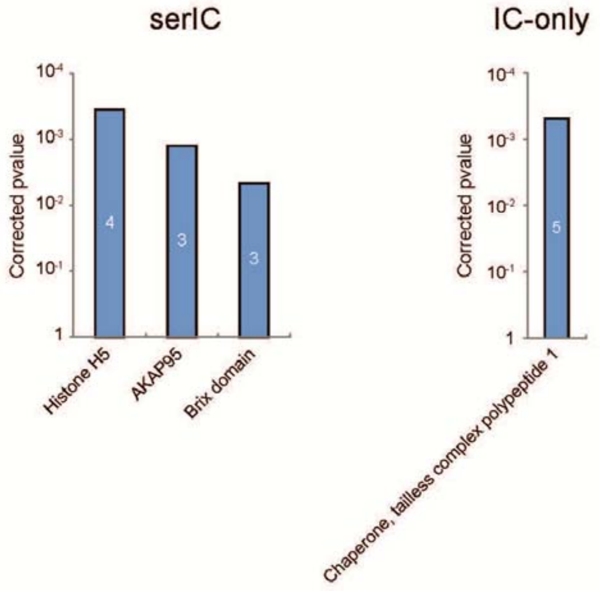

Supplementary Figure 4: Domains in no-RBD RBPs.

(**a-b**) Shown are protein domains that are enriched within serIC-derived (**a**) or IC-only (**b**) no-RBD proteins with a corrected pvalue below 0.05 (Benjamini-Hochberg).

Supplementary Figure 5

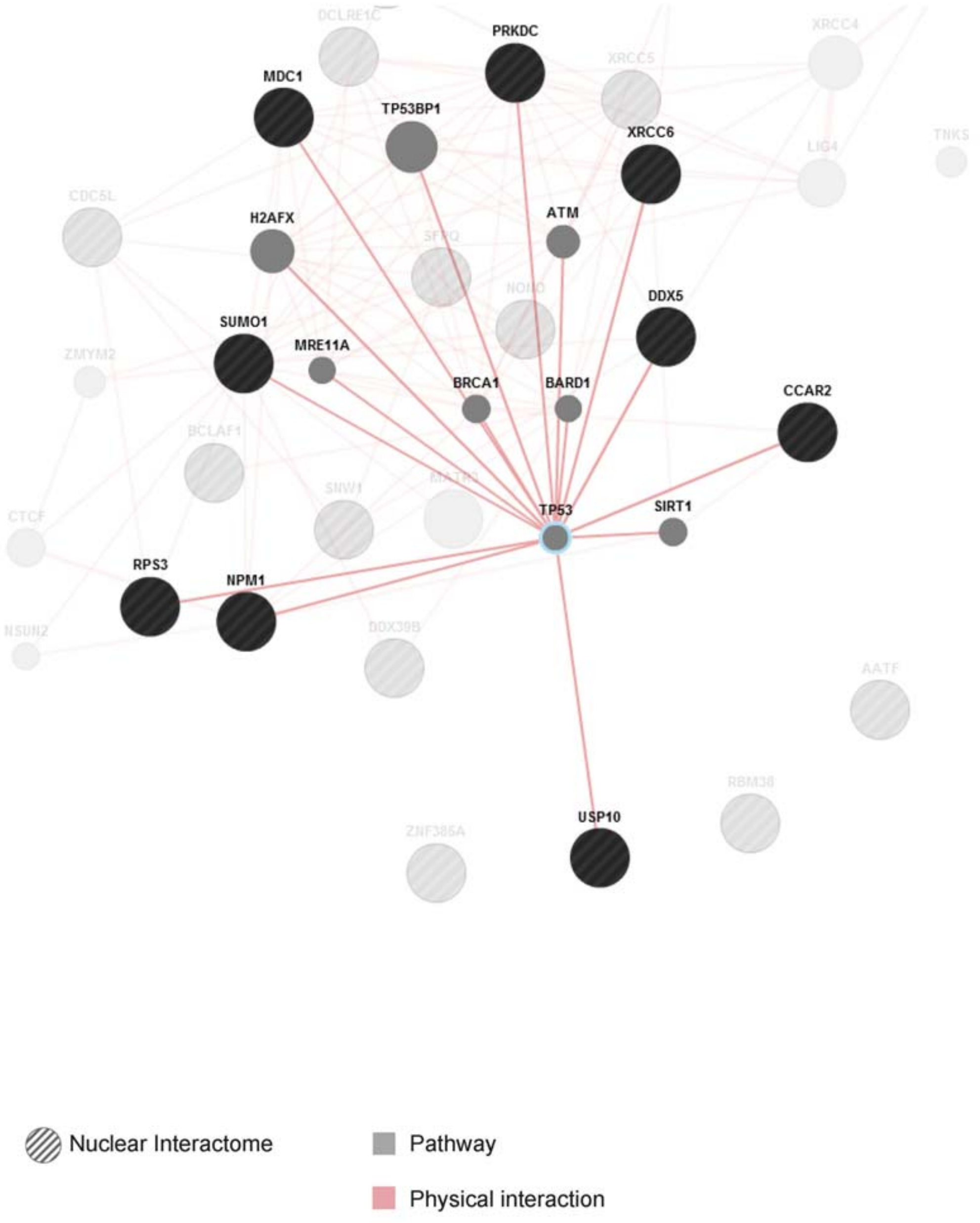

Supplementary Figure 5: The TP53 interacting nuclear interactome.

Same network as in Figure 5 with physical binding partners of TP53 highlighted.

Supplementary Figure 6

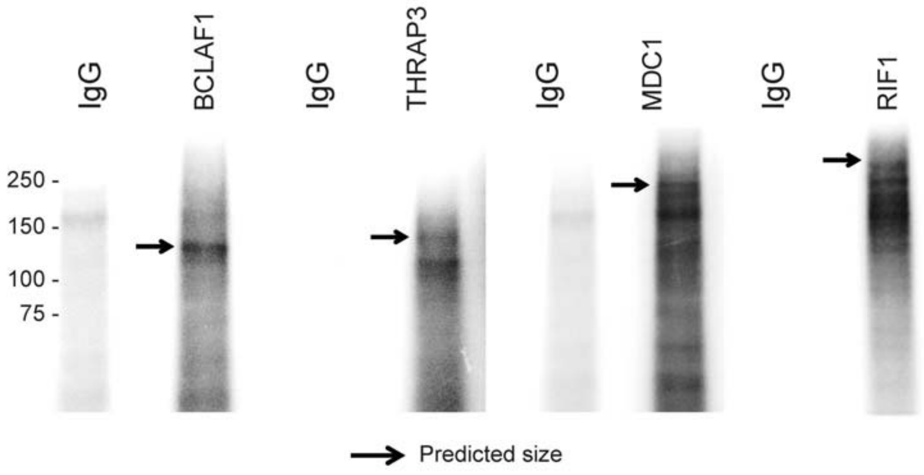

Supplementary Figure 6: Validation of protein-RNA interaction of novel RBPs.

The indicated endogenous proteins were immunoprecipitated from UV-crosslinked K562 cells using antibodies. Crosslinked RNA was radiolabeled with PNK and 32P-ATP, and residual DNA was digested after the labelling step with turbo DNAse. Protein-RNA complexes were separated by PAGE and crosslinked RNA was visualized by exposure to a phosphoimager screen.

Supplementary Methods

CLIP

Cells were irradiated with UV-C light at 254nm, lysed in RIPA buffer (50mM Tris pH 7.4, 150mM NaCl, 1mM EDTA, 0.5% Igepal, 0.5% DOC, 0.1% SDS) and immunoprecipitated using 2µg of rabbit anti-BTF/BCLAF1 (Bethyl A300-610); rabbit anti-THRAP3 (Bethyl A300-955A); rabbit anti-MDC1 (Bethyl A300-053A); or rabbit anti-RIF1 (Bethyl A300-567A). Immunocomplexes were washed three times with RIPA-HS (50mM Tris pH 7.4, 600mM NaCl, 1mM EDTA, 0.5% Igepal, 0.5% DOC, 0.1% SDS) and crosslinked RNA was trimmed by RNAseI. Protein-RNA complexes were separated by PAGE and visualized by exposure to a phosphoimager screen.
